# Continuous and simultaneous estimation of finger kinematics using inputs from an EMG-to-muscle activation model

**DOI:** 10.1186/1743-0003-11-122

**Published:** 2014-08-14

**Authors:** Jimson G Ngeo, Tomoya Tamei, Tomohiro Shibata

**Affiliations:** Graduate School of Information Science, Nara Institute of Science and Technology, Nara, Japan; Graduate School of Life Science and Systems Engineering, Kyushu Institute of Technology, Kitakyushu, Japan

**Keywords:** Surface Electromyography (EMG), Muscle activation model, Finger kinematics, Neural networks, Gaussian process regression

## Abstract

**Background:**

Surface electromyography (EMG) signals are often used in many robot and rehabilitation applications because these reflect motor intentions of users very well. However, very few studies have focused on the accurate and proportional control of the human hand using EMG signals. Many have focused on discrete gesture classification and some have encountered inherent problems such as electro-mechanical delays (EMD). Here, we present a new method for estimating simultaneous and multiple finger kinematics from multi-channel surface EMG signals.

**Method:**

In this study, surface EMG signals from the forearm and finger kinematic data were extracted from ten able-bodied subjects while they were tasked to do individual and simultaneous multiple finger flexion and extension movements in free space. Instead of using traditional time-domain features of EMG, an EMG-to-Muscle Activation model that parameterizes EMD was used and shown to give better estimation performance. A fast feed forward artificial neural network (ANN) and a nonparametric Gaussian Process (GP) regressor were both used and evaluated to estimate complex finger kinematics, with the latter rarely used in the other related literature.

**Results:**

The estimation accuracies, in terms of mean correlation coefficient, were 0.85±0.07, 0.78±0.06 and 0.73±0.04 for the metacarpophalangeal (MCP), proximal interphalangeal (PIP) and the distal interphalangeal (DIP) finger joint DOFs, respectively. The mean root-mean-square error in each individual DOF ranged from 5 to 15%. We show that estimation improved using the proposed muscle activation inputs compared to other features, and that using GP regression gave better estimation results when using fewer training samples.

**Conclusion:**

The proposed method provides a viable means of capturing the general trend of finger movements and shows a good way of estimating finger joint kinematics using a muscle activation model that parameterizes EMD. The results from this study demonstrates a potential control strategy based on EMG that can be applied for simultaneous and continuous control of multiple DOF(s) devices such as robotic hand/finger prostheses or exoskeletons.

**Electronic supplementary material:**

The online version of this article (doi:10.1186/1743-0003-11-122) contains supplementary material, which is available to authorized users.

## Introduction

Robotic hand assistive devices and tele-manipulation devices are developing technologies that hold great promise in revolutionizing modern hand rehabilitation and prosthetic application. Today, many such robotic hand prosthesis devices and exoskeletons with many degrees-of-freedom (DOF) have been and are continuously being developed. Roughly about 30% to 50% of the available prosthesis is based on myoelectric control [1].

Tele-operated devices controlled by neural signals can give unconstrained and precise movement control in different environments [2]. Surface electromyogram (EMG) signals are often used in prosthesis controls and rehabilitation support applications because these reflect the motor intention of a user prior to the actual movements [3]. These signals not only provide little delay when used in human interfaces, but have also been shown to represent muscle tension and joint positions very well.

In the past 30 years, discrete classification of hand gestures from EMG signals has been successful, consistently reaching decoding accuracies of above 95% and classifying more than 6 gestures, such as hand opening, closing and others [4, 5]. However, classification approaches have been limited to use in controlled laboratory conditions and have not been used by any current commercial myoelectric prosthesis [6].

Despite the success of pattern classification approach to EMG signals, this type of control strategy is inadequate for actuating all the functions offered by the robotic device as it uses a sequential strategy where only one class of movement is active at a time [7]. Also, natural hand movements are not limited to discrete gestures but are continuous, coordinated and have simultaneous control of multiple DOFs.

To realize a more intuitive and natural myoelectric control scheme, control strategies based on proportional and simultaneous control are preferred over discrete classification based control. This study aims to estimate simultaneous and multiple finger kinematics from surface EMG signals.

An example of simultaneous control of multiple DOFs was shown by Jiang et al. using muscle synergy strategies extracted from a modified nonnegative matrix factorization algorithm to estimate the torque [8] and kinematics [9, 10] of multiple DOFs produced at the wrists. Relating to finger-based applications, studies have shown that it is possible to extract fine finger movement information contained in surface EMG signals. Afshar and Matsuoka [2] were able to estimate the index finger joint angles from fine-wire EMG embedded inside seven muscles that control the index finger. Similarly, Shrirao et al. [11] were able to decode one index finger joint angle from surface EMG signals. The finger motions involved in their study were periodic flexion-extension movements at three different speeds and they evaluated many different committees of neural network but failed to get a consistent robust optimal configuration. Furthermore, Smith et al. [12] were able to asynchronously decode individual metacarpophalangeal (MCP) joint angles of all five fingers using an artificial neural network. Their study extracted time-domain features from 16 general muscle locations in healthy subjects. However, the number of channels involved may be too many for practical applications, and movements were limited to moving only one finger at a time despite simultaneous recording. In more recent developments, Hioki et al. [13] estimated five proximal interphalangael (PIP) joint angles using only four EMG channels and considered the dynamical relationship between the EMG and the finger actuation by adopting time delay factors and feedback stream into an artificial neural network. Their method, however, has complex parameter configuration wherein the number of parameters drastically varies with different settings. In the previous studies [11–13], a time delay between the onset of the EMG signal and the exerted movement was present and observed. This time delay is called hysteresis or electromechanical delay (EMD). Introducing EMG-tapped delay lines, which makes use of all the immediate and past values of the EMG can consider for this delay. However, doing so greatly increases the dimension of the inputs and thus exponentially increases the number of parameters of the regressor used. EMD can vary depending on many different factors such as muscle shortening velocity, type of muscle fiber, and fatigue [14].

The present study aims at overcoming the above limitations by introducing EMD as a parameter, by using a so-called EMG-to-muscle activation model [3, 14, 15], which is determined along with other system parameters through optimization. Very few studies have continuously estimated more than five finger positions, but here we present a method for the continuous extraction of control information during finger movements which involves simultaneous activation of 15 DOFs provided by all five finger joints. We concurrently recorded the kinematics of all five fingers in one hand and the surface EMG signals from muscles in the forearm while the subjects performed both individual and simultaneous finger flexion and extension tasks. Simultaneous estimation of the finger kinematics is done and evaluated using both a fast feedforward artificial neural network and a nonparametric Gaussian Process regression [16], with the latter having the potential to give better estimation performance but rarely used in myoelectric control literature.

This paper describes a new strategy to estimate complex finger kinematics that can be used to augment current myoelectric prosthetic control schemes. Simultaneous and multiple finger joint positions, namely the metacarpophalangeal (MCP), proximal interphalangael (PIP) and the distal interphalangeal (DIP) joints of all 5 fingers in a hand are mapped from EMG signals using a model-free based approach which involves the use of machine learning regression techniques.

## Methods

### Participants

Similar to contemporary studies that proposed new EMG-based control strategies for hand control [5, 7, 11–13], healthy, able-bodied subjects participated in the experiments, which can be an initial basis before testing with disabled or amputated subjects. Ten healthy participants (7 male, 3 female, aged 27 ±4 years), who gave informed consent to participate in the experiment protocol, volunteered in this study. The participants had no previous experience with myoelectric control nor with any 3D motion capture experiments.

### Experimental setup

The system is mainly composed of a wireless multi-channel surface electromyograph and a 3D optical motion capture device. Surface EMG signals, as well as the kinematics of unrestrained and continuous hand and finger movements, were simultaneously recorded.

#### EMG recording

For all the subjects, surface EMG signals were extracted from eight extrinsic muscles of the forearm that are known to contribute to wrist and finger movements. Four flexor muscles and four extensor muscles in the forearm were targeted. These target muscles along with their corresponding function related to any hand or finger movements are listed in Table 1.

**Table 1:** **Selected EMG channels and the target muscles**

Channel	Target muscle	Hand/Finger
1	Abductor pollicis longus	Thumb abduction
2	Flexor carpi radialis	Wrist, hand flexion and abduction
3	Flexor digitorum superficialis	2-5th finger PIP flexion
4	Flexor digitorum profundus	2-5th finger DIP flexion
5	Extensor digitorium	2-5th finger extension
6	Extensor indices	Index finger
7	Extensor carpi ulnaris	Wrist extension and abduction
8	Extensor carpi radialis	Wrist and thumb

Source: Anatomy and Kinesiology of the Hand [17].

**Figure 1:**
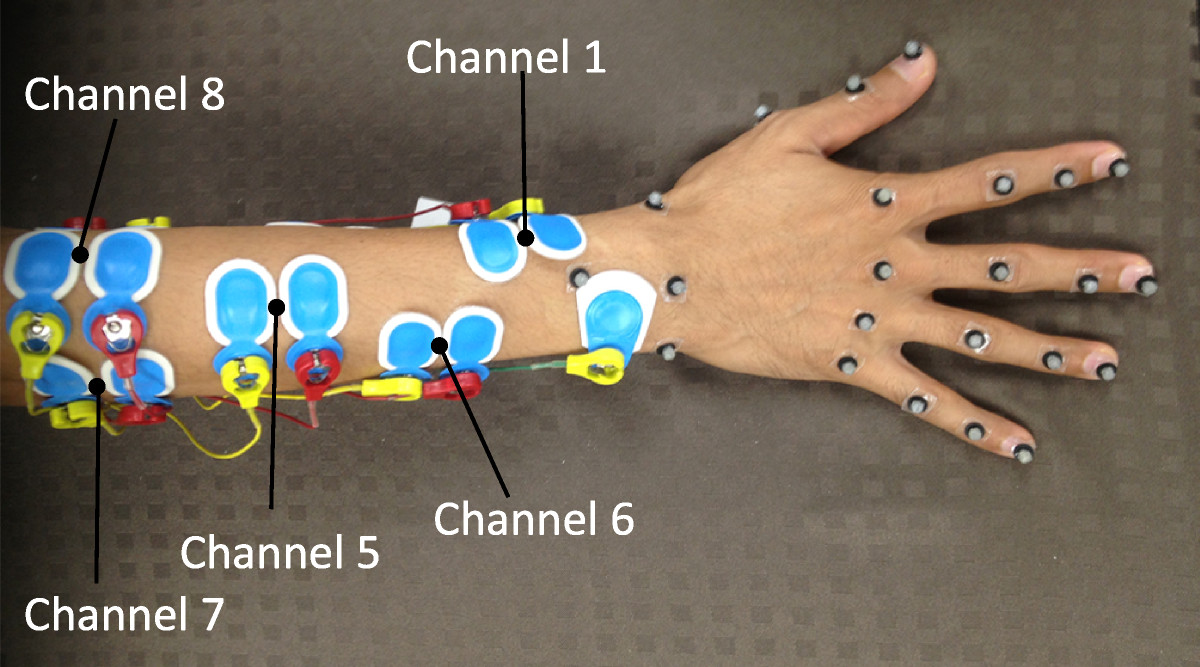
**EMG placement.** The surface EMG setup and the general view of EMG placement on a subject. The corresponding target muscle for each channel is shown in Table 1.

Eight bipolar active-type Ag-AgCl electrodes from Ambu, with an average inter-electrode distance of 20 mm were placed on the the subjects as shown in Figure 1. The target muscles were mostly found by palpation, anatomical landmarks described in [17], and by visual inspection of the signal that gave the best response to describe the movements listed in Table 1. A single electrode was also placed on the subject’s olecranon to serve as a ground and reference electrode. The surface electrodes were connected to a BA1104 pre-amplifier with a telemetry unit TU-4 (Digitex laboratory co. ltd.). The hardware provided a low-pass filter with cut-off frequency of 1 kHz during the EMG data acquisition process. The EMG signals were sampled at 2 kHz, and were digitized by an A/D converter with 12-bit precision. The EMG signals were displayed on a real-time monitor and visually inspected to ensure quality of the signal.

#### Finger kinematics recording

**Figure 2:**
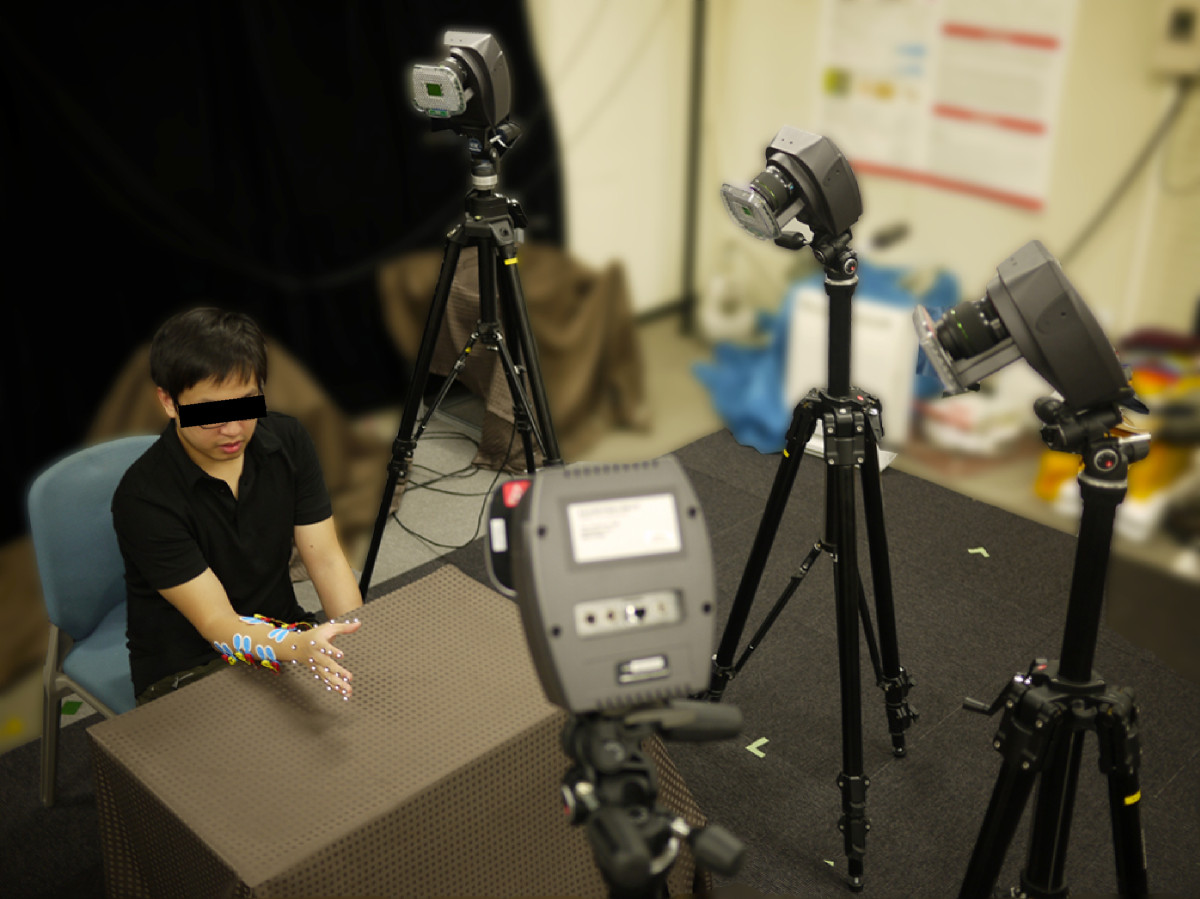
**Camera Set-up.** The overview of the 3D motion camera system.

**Figure 3:**
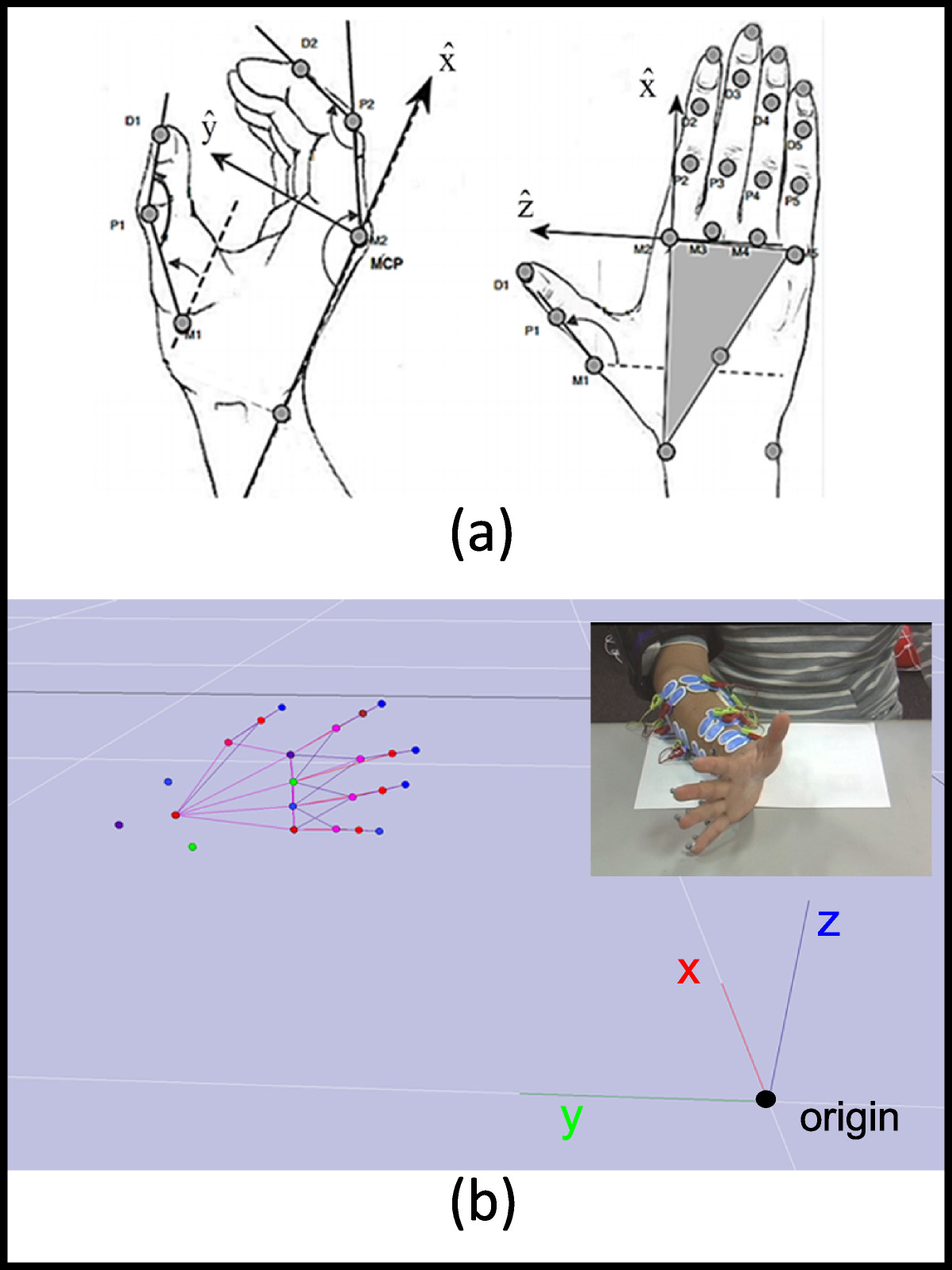
**Marker locations on the finger joints. (a)** Twenty-two markers are attached on the hand. **(b)** Hand skeleton model on the motion capture system.

While finger movements were made, the hand and finger motion were recorded simultaneously using a MAC3D motion capture system (Motion Analysis Corp.). The camera set-up using the mounted Eagle cameras is shown in Figure 2. Twenty-two passive reflective markers for motion capture were attached on the subject’s hand, with a marker located on each joint of the finger and three in the wrist area (see Figure 3). Small 6-mm diameter markers were used to reduce switching marker errors and to avoid getting the markers too close to each other. The optical cameras were positioned and calibrated to capture a volume (500×700×500 mm) space that would be able to effectively see and measure the small markers. The Cortex software from Motion Analysis was used to concurrently record the EMG and motion data. A sample skeleton model used in the marker data acquisition is shown in Figure 3.

The marker trajectories were sampled at 200 Hz with measurement units in millimeters, having residual errors of less than 0.5 mm (as indicated during the Cortex calibration procedure). With the x, y, z positions of each marker continuously recorded, the joint positions, namely the MCP, PIP, and DIP joint angles, were calculated. Because the thumb does not have a DIP joint, the carpometacarpal (CMC) joint was considered before the MCP joint.

### Data collection

The participants were individually seated on a regular chair, with their hand and elbow comfortably positioned on a flat surface table centered on the motion capture volume area. Each subject was tasked to do 3 different tasks. For the first part of the experiment protocol, the subject was tasked to move one finger at a time, in the flexion-extension plane of each finger. The second task involved the subject moving all fingers simultaneously in the same flexion-extension plane. This motion resembled the opening and semi-closing of the hand. Full closing of the hand was not possible as some markers at the tip of the fingers would not be seen by the motion capture system. In these first two tasks, the subjects mainly did MCP flexion and extension, in which the PIP and DIP followed the movements of the MCP joint. Finally for the third and last part of the experiment, the subject was tasked to move any finger freely in any direction within the motion capture volume space while still maintaining a fixed neutral position for the arm and elbow. Irregular movements and different finger combinations for flexion and extension movement were encouraged from the subject in this last part of the experiment.

In any of the trials, the subjects were allowed to make as many movements as they wanted to but were asked to move in their own perceived normal velocity (which did not exceed 2 cycles of flexion and extension movement per second). The subjects were tasked to reach maximum flexion and extension for each finger at least once at any point in any of the trials. All the movements were limited to finger flexion and extension movements while the rest of the arm (e.g. wrist, elbow, etc.) maintained a fixed position upon instruction. Markers on the wrist joint were also recorded to ensure that the wrist maintained a fixed position, or at least minimal ulnar/radial angle deviation.

The first task consisted of 5 sets of movements, one for each individual finger. While the second task consisted of 2 sets and the last task consisted of 1. Overall, the whole experiment consisted of making 8 sets of movement tasks. In each set, there were 5 trials, with each trial lasting 20 seconds. All trials were sequentially done and the subjects were allowed to rest anytime throughout the experiment. The subjects were also instructed to, as much as possible, maintain the position of the whole arm in a neutral and relaxed position while the fingers were moving, to reduce fatigue.

After collecting the EMG data along with the motion capture of the finger movements, separate trials were also done to obtain a maximum voluntary contraction (MVC) of each muscle. The subjects were asked to flex their hands and fingers in all possible planes of movement to try and induce maximum contractions for all the targeted muscles in the forearm. However, it is very hard to obtain the true maximum EMG values, so we instead obtained the maximum rectified EMG value from all the trials including the separate trials for obtaining the MVC of each muscle.

Eighty percent of all the recorded data were used for training and validation and the remaining twenty percent were used for testing. All the data in each task were concatenated together to form a larger training and test dataset. However, the data were separated and were analyzed separately for each subject.

### Data processing

#### EMG-to-muscle activation model

For any intended motor action, it is known that there occurs a time delay, which is known as the electromechanical delay (EMD), between the onset of the EMG signals and the exerting tension in the muscles. EMD has been observed by previous studies in the leg and as well as in the arm [11, 14, 18]. EMD has been reported to range from 10 ms to about 150 ms, but varies differently depending on the intended tasks [3]. Thus, EMD cannot be ignored in EMG studies involving motor actions, and must be considered accordingly. In this study, through visual inspection and by applying Fast Fourier Transform (FFT) on the motion data, it was verified that the highest frequency of any periodic finger flexion and extension movement did not exceed 2 Hz.

The raw EMG signals were first preprocessed into a form, that after further manipulation, can be used to estimate muscle activation [14]. The EMG signals were then rectified, normalized by dividing by the peak rectified EMG obtained, and low-pass filtered (4 Hz cut-off frequency, zero-phase 2nd-order Butterworth filter). This is done prior to obtaining the muscle activations, which are highly related to muscle force found in low frequencies [14]. The filtered EMG signals were then downsampled to 200 Hz to match that of the motion data.

To learn a suitable filtered signal which automatically considers EMD, we introduce the use of a so-called EMG-to-Muscle activation model, in place of using EMG tapped-delay lines. EMG is a measure of electrical activity that spreads across muscles, which causes the muscles to activate. This results to the production of force, where the model transforms the EMG signals to a suitable force or muscle activation representation.

Zajac modeled the muscle activation dynamics using a first-order recursive filter [15]. Although a first-order differential equation does a fine job of characterizing activation, Buchanan et al. created a second-order model filter that works efficiently to model the relationship between EMG and muscle activation [14]. In this study, we make use of the filter in an approximate discrete function given by:

1 $$\begin{array}{l} u_{j}(t)=\alpha e_{j}(t-d)-\beta_{1}u_{j}(t-1)-\beta_{2}u_{j}(t-2) \end{array}$$

where *e*_*j*_(*t*) is the normalized, rectified and filtered EMG of muscle *j* at time *t*. In this model, *α*, *β*_1_ and *β*_2_ are recursive coefficients of the filter and *d* is the EMD. Filter stability is guaranteed by subjecting *α*, *β*_1_, and *β*_2_ to the following constraints:

2 $$\begin{array}{l} \beta_{1}=\gamma_{1}+\gamma_{2}\; \end{array}$$

3 $$\begin{array}{l} \beta_{2}=\gamma_{1}\cdot \gamma_{2}\; \end{array}$$

4 $$\begin{array}{l} \left|\gamma_{1}\right|<1,\left|\gamma_{2}\right|<1\; \end{array}$$

5 $$\begin{array}{l} \alpha -\beta_{1}-\beta_{2}=1\; \end{array}$$

In this model, neural activation *u*(*t*) depends not just on the current level of EMG but also on its recent history and is constrained from 0 to 1. Studies have also shown that while some muscles have linear isometric EMG-to-force relationship, the relationship for other muscle conditions are nonlinear in nature [14]. This nonlinearity between muscle activation *v*_*j*_ and its neural activation *u*(*t*) can be modelled as:

6 $$\begin{array}{l} v_{j}=\frac{e^{A_{j}u_{j}(t)}-1}{e^{A_{j}}-1} \end{array}$$

where *A*_*j*_ is constrained between -3 and 0, with -3 being highly exponential and 0 being linear.

Using this muscle activation model not only solves the EMD of the muscle, but also requires only a few parameters. The parameters of this filter, *γ*_1_, *γ*_2_, *d*, and *A* are obtained by using constrained nonlinear programming in Matlab Optimization Toolbox (Mathworks, Inc.) to minimize a mean-square error cost function given by:

$$\begin{array}{l} \frac{1}{N}\underset{t}{\sum }{\left(\theta_{\text{est}}-\theta_{\text{target}}\right)}^{2} \end{array}$$

where *N* is the total number of samples, and *θ*_*est*_ and *θ*_*target*_ are the estimated and measured finger joint angles, respectively.

#### Other EMG features

Because of the great success in movement classification from myographic signals, time-domain features have been extensively used. To show that the finger kinematic estimation performance was better using the proposed muscle activation model that considers electromechanical delay, we used four conventional time domain (TD) features, namely the Mean of the Absolute Value (MAV), Waveform Length (WL), Willison Amplitude (WA) and Variance (VAR) [19, 20]. These features provide different information such as those pertaining to signal amplitude, frequency, extent of muscle contraction, and extent of the firing of motor unit action potentials. The length of the sliding window was 200 ms with a 25 ms overlap. In the preliminary investigation (not reported) of this study, other time and frequency domain features gave high correlation with the four features used and did not provide better estimation performance. These features were also used by most of the previous studies that performed finger joint kinematic and dynamic estimation from EMG [2, 11–13].

#### Hand/Finger kinematics

Each of the five fingers produced all three joint angles of interest. The tasks were constrained to moving the fingers only in the flexion and extension plane, thus, a total of 15 DOFs were considered. The joint angles were computed from the recorded marker trajectories. A low-pass filter with cut-off frequency of 10 Hz was also applied on the motion data, to remove any noise and jitters in the signal.

The range of motion given for each of the 15 DOFs is presented in Table 2 taken from the average of all the subjects. These were based from the minimum and maximum value of the computed joint angle kinematics. Table 2 reflects the variability in range of finger motions that the subjects are capable of. Attributes such as the physical lengths and widths of the finger joints contributed to the change in range of motions.

**Table 2:** **Finger joints normal range of motion**

Finger joint	DOF	Type of motion	Theoretical range	Measured range
Thumb CMC	1	Hyperextension/Flexion	-10/55 deg	9.86±21.17/50.06±11.39 deg
Thumb MCP	2	Hyperextension/Flexion	-10/55 deg	-3.05±4.94/56.51± 8.34 deg
Thumb IP	3	Hyperextension/Flexion	-15/80 deg	-4.52±8.40/57.27±18.01 deg
Index MCP	4	Extension/Flexion	-45/90 deg	-39.97±15.00/62.29±14.27 deg
Index PIP	5	Extension/Flexion	0/100 deg	-14.95±12.42/72.55±16.87 deg
Index DIP	6	Extension/Flexion	0/80 deg	-16.96±13.97/45.51±22.25 deg
Middle MCP	7	Extension/Flexion	-45/90 deg	-34.07±10.29/69.39±11.61 deg
Middle PIP	8	Extension/Flexion	0/100 deg	-16.87±12.88/80.07±16.52 deg
Middle DIP	9	Extension/Flexion	0/80 deg	-15.15±11.77/57.07±22.42 deg
Ring MCP	10	Extension/Flexion	-45/90 deg	-26.35±10.71/62.51±11.04 deg
Ring PIP	11	Extension/Flexion	0/100 deg	-15.34±11.44/88.58±14.21 deg
Ring DIP	12	Extension/Flexion	0/80 deg	-14.52±11.36/58.94±19.99 deg
Little MCP	13	Extension/Flexion	-45/90 deg	-14.31±12.59/69.27± 6.07 deg
Little PIP	14	Extension/Flexion	0/100 deg	-14.66±12.59/72.94±14.27 deg
Little DIP	15	Extension/Flexion	0/80 deg	-10.09±8.45/84.54±12.60 deg

In the regression step, however, to standardize and scale all the joint angle values, we normalized each finger DOF to show a scaled value from 0 to 1. Normalization of each joint angle data was done by subtracting the minimum of the joint angle to each sample and dividing it by the difference between the maximum and minimum measured joint angle.

#### Feedforward artificial neural network

In general, neural networks (NN) are considered to be attractive for nonlinear modelling because of its ability to approximate any arbitrary function [21]. A multi-layer feed forward neural network was used to learn a mapping between the EMG signals and the corresponding hand/finger kinematics. All 15 DOFs of the fingers were simultaneously and continuously estimated using the neural network:

7 $$\theta_{\text{est}}(t)=\text{NN}(v(t),w)$$

where **θ**_*est*_(*t*)∈**R**^15×1^ is the estimated finger joint angle, **v**(*t*)∈**R**^8×1^ is the muscle activation input, and **w** contains the weight parameters which represent the links between the nodes or neurons. The network is made up of an input layer, a hidden layer with a tan-sigmoidal activation function, and a single linear output layer. The neural networks were implemented using the Netlab toolbox [22]. Parameters of the network were obtained by minimizing a mean square error function. The network’s performance was evaluated with various numbers of neurons in the hidden layer, ranging from 5 to 350. Using a fixed training set, we chose the specific number of neurons in the hidden layer based on which solution gave the smallest average error on an unseen test set. To avoid overfitting, only 80% of the total dataset was used for training and validation and an early stopping method was applied during training iterations [23].

#### Gaussian process for regression

Neural networks are used in almost all studies related to human kinematics and kinetics estimation from EMG despite the fact that its structure is heuristic and gives a “black box” model approach to estimation. However, the choice of a neural network is justified as this gives very fast computation time even when estimating in an online fashion. In this study, we wanted to verify if using a nonparametric Bayesian regressor could greatly improve estimation performance [24]. Recently, more popular nonparametric Bayesian approaches such as the Gaussian Process (GP) Regression have gained attention in being able to improve estimation performance in certain cases. GP regression is fundamentally different from feed-forward networks. Rather than capturing regularities in the training data via updating neuron weights, it applies a Bayesian inference to explicitly compute a posterior distribution over possible output values *y* given all the data and the new input *x*[16, 25]. In this study, a GP regressor was used and evaluated:

8 $$\theta_{\text{est}}(t)=\mathrm{GP}\left(m(v),k\left(v,v^{\prime }\right)\right)$$

where **v** is the muscle activation input, **θ**_*est*_ is the estimated kinematics and the Gaussian process is determined by a mean function *m*(**v**) and covariance function *k*(**v**,**v**^′^).

The GP regression was implemented using Gaussian Process Regression and Classification Toolbox [26]. The muscle activation training data were standardized to have zero mean and unit variance on each dimension while the test data were standardized to have its mean centered around the training mean. This standardized data was also used for the neural network for an objective comparison between the two regression methods. The GP configuration assumed a zero mean function, a Gaussian likelihood function (with one hyperparameter *σ*_*n*_), and a squared exponential covariance function (with two additional hyperparameters: a characteristic length-scale *l* and unit signal standard deviation *σ*_*f*_) [16].

9 m(v)=0

10 $$k\left(v,v^{\prime }\right)=\sigma_{f}^{2}\;\text{exp}\left[\frac{-{\left(v-v^{\prime }\right)}^{2}}{2\;l^{2}}\right]$$

The reliability of the GP regression is highly dependent on the chosen covariance function. A maximum posterior estimate of the hyperparameters **w** (e.g. **w**={*σ*_*f*_,*σ*_*n*_,*l*}) occurs when the posterior probability *p*(**w**∣**x**,**y**) is at its greatest. Baye’s theorem tells us that, assuming there is little prior knowledge about what **w** should be, this corresponds to minimizing the negative log likelihood given by:

11 $$\begin{array}{l} \text{ln}\;\;p(\theta |v,w)=-\frac{1}{2}\theta^{⊤}K^{-1}\theta -\frac{1}{2}\text{ln}|K|-\frac{N}{2}\text{ln}\;2\pi \end{array}$$

An exact inference method was used and optimized to get good choices for **w**[16].

Unlike in the use of the artificial neural network where one network produced all 15 joint angle outputs simultaneously, a dedicated GP regressor was created for each DOF. In the training stage, 15 GP regressors were individually trained and then used to estimate simultaneous movements of all 15 DOFs. Also, because the learning from the log-likelihood involves the computation of the inverse of *K*, which is the covariance matrix whose complexity grows as the size of the input or output matrix increases. We used a fixed interval sampling to reduce the number of training samples which significantly reduces the hyperparameter learning and training time needed.

#### Statistical analysis

A five-fold cross validation procedure was used to evaluate the overall statistical performance of the two different estimators and when different input features were used. Two performance indices were chosen to evaluate how accurately each finger DOF was estimated. The Pearson’s correlation coefficient or the *R*-value index describes the total variation between the actual and estimated samples, while the normalized root-mean square error (NRMSE) describes the total residual error. These two performance indices are defined as the following:

12 $$\begin{array}{ll} R_{i} & \;=\;\frac{\sum_{t=0}^{N}\left(\theta_{\text{act}}\;-\;\mu_{\text{act}}\right)\left(\theta_{\text{est}}\;-\;\mu_{\text{est}}\right)}{\sqrt{\sum_{t=0}^{N}{\left(\theta_{\text{act}}\;-\;\mu_{\text{act}}\right)}^{2}}\sqrt{\sum_{t=0}^{N}{\left(\theta_{\text{est}}\;-\;\mu_{\text{est}}\right)}^{2}}}\; \end{array}$$

13 $$\begin{array}{ll} {\text{NRMSE}}_{i} & \;=\;\sqrt{\frac{\sum_{t=0}^{N}{\left(\theta_{\text{act}}-\theta_{\text{est}}\right)}^{2}}{N}}\; \end{array}$$

where *θ*_*act*_ and *θ*_*est*_ are the normalized actual measured and estimated DOFs, respectively, *μ* represents the mean and *R*_*i*_ and NRMSE_*i*_ are the correlation coefficient and normalized root-mean-square error of the *i* th DOF, respectively.

Three different statistical analysis procedures were made in this study. A three-way analysis of variance (ANOVA) was done to compare the effects of different factors on the global estimation performance when NN regression was used. The different factors that we considered were the subject (S1-S10), the finger DOFs (15 DOF) and the type of input feature (filtered EMG, TD-based or muscle activation) used. When significant interaction was detected, focused ANOVA was conducted by fixing the levels of one of the interacting factors [10]. When no interaction was detected, a reduced ANOVA model with only the main factor was performed. Tukey-Kramers post-hoc comparison test was performed when significance was detected. The second procedure was a one-way ANOVA followed by the same post-hoc comparisons, which was used to compare any significant differences in the obtained parameters, such as the EMD between subjects. The third and final one was to investigate the effects of using different regression models or methods (such as GP versus NN) on the global estimation performance. Separate t-tests and ANOVA were used for this procedure. The significance level was set to 95% and all the procedures mentioning the global estimation performance were performed on results of the test sets.

## Results

**Figure 4:**
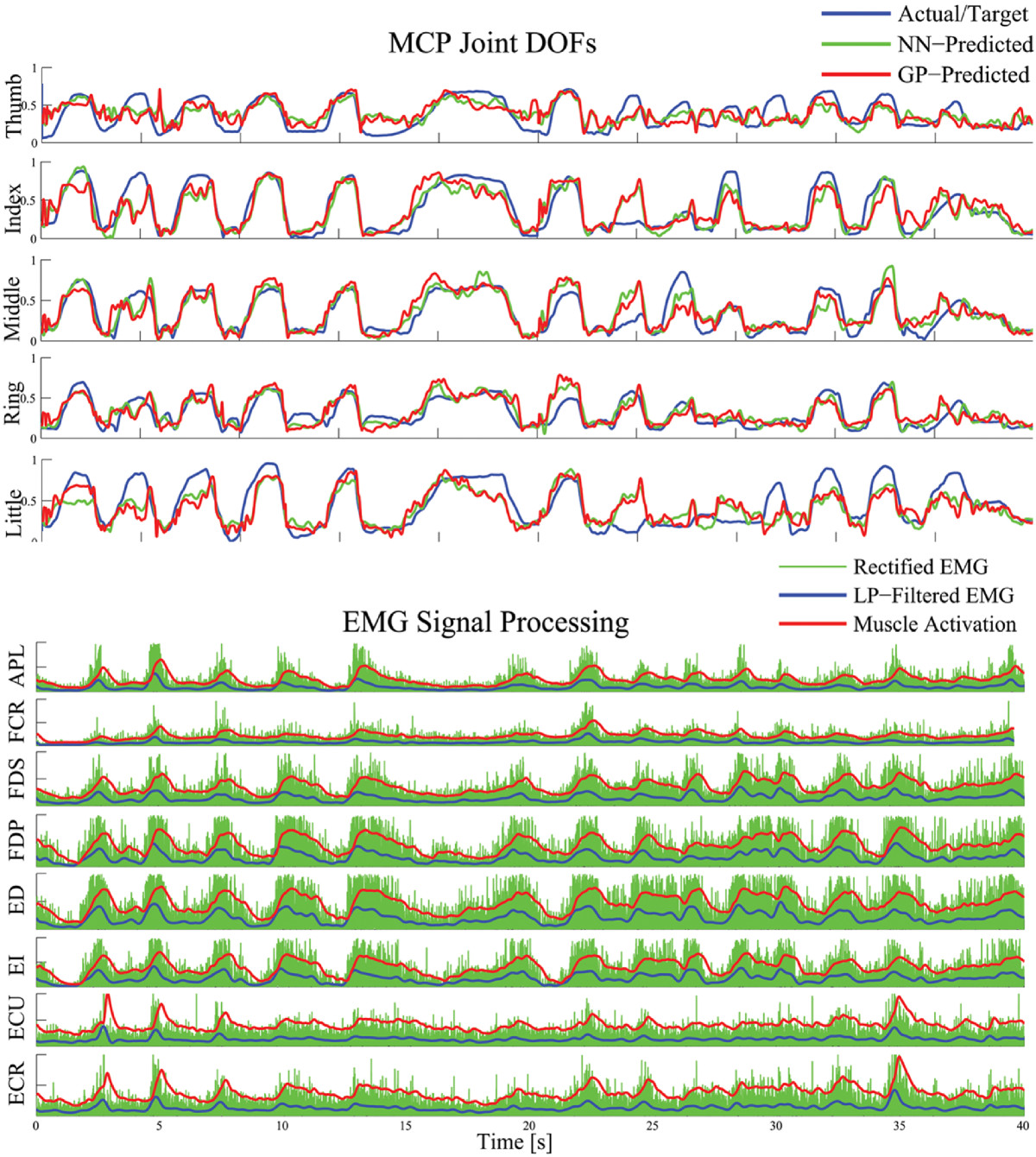
**One representative subject data.** 5 out of the 15 normalized finger joint angles are shown in blue solid lines, while the neural network (NN) and Gaussian Process (GP) estimated results are shown in green and red, respectively. Below the joint angle results are the 8-channel processed EMG from the subject which includes the following: rectified EMG (green), low-pass filtered EMG (blue) and the transformed muscle activations (red). In this test data, the parameters obtained for the muscle activation model were: *A*=-3, *d*=0.045, *γ*_1_=*γ*_2_=-0.9539. The labels on the y-axis of the plots correspond to the target EMG channels which is listed in Table 1.

With the neural network and Gaussian Process regressors trained, all 15 finger DOFs were estimated simultaneously. Figure 4 shows a representative estimation result taken from 1 test trial from a subject. In this result, the subject performed simultaneous joint flexion and extension of all finger joints. Though only the MCP finger joint angles are shown in the figure, the PIP and DIP angles showed consistent results with the MCP angles since this task involved the flexion and extension of all joints simultaneously. The NN and GP regressors were trained with 4800 samples. The average correlation coefficient of the GP-estimated results were significantly higher than the NN-estimated results (*R*, 0.84±0.0378 versus 0.71±0.0981; *P*<0.001). With more training samples used, correlations between the actual and estimated value for a single DOF reached as high as 0.92 for the MCP joint angle estimation. While the DOFs for the smaller finger PIP and DIP joints reached as high as about 0.85 and 0.79 in correlation, respectively.

**Figure 5:**
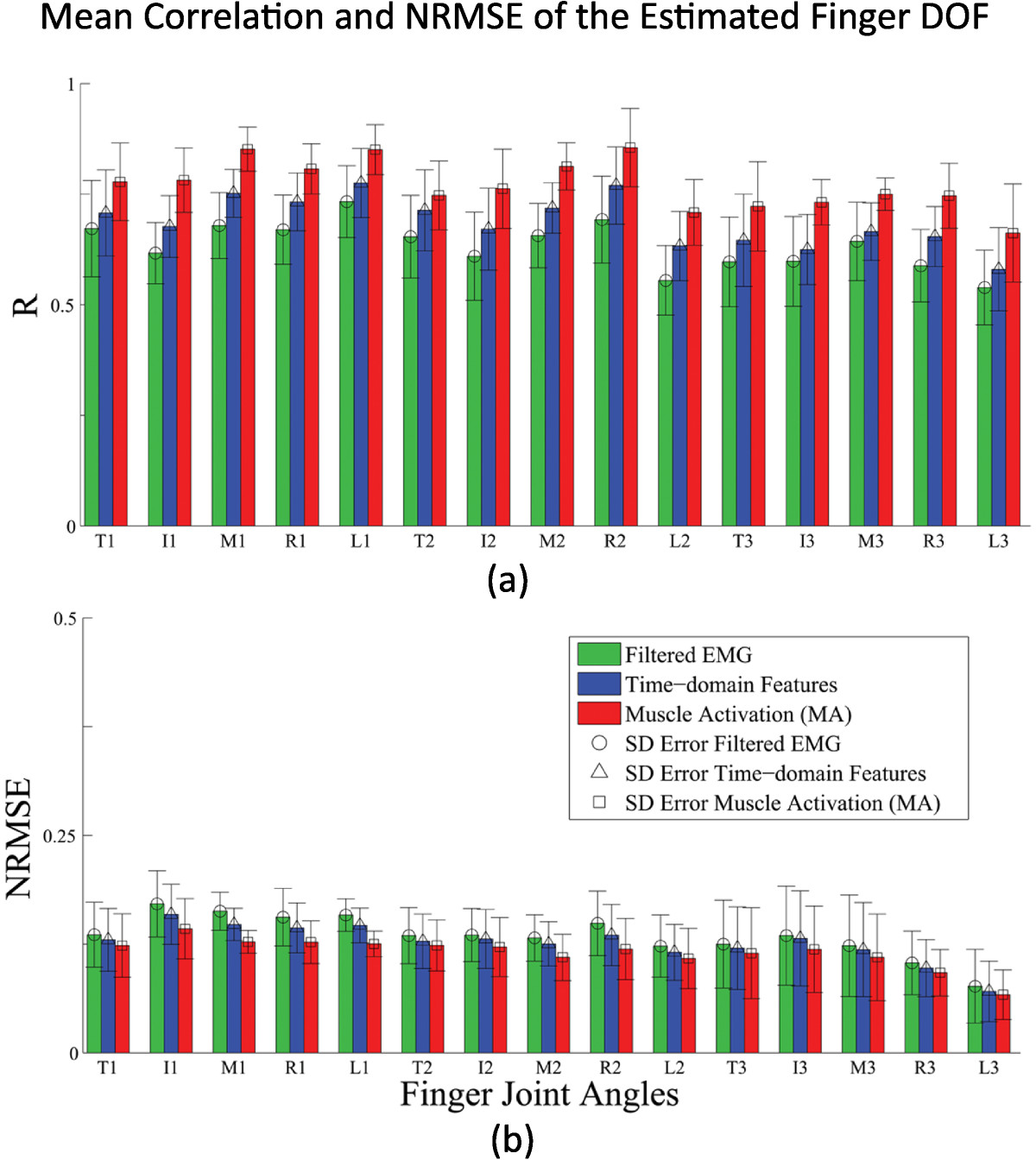
**Overall estimation performance.(a)** The mean correlation coefficient and **(b)** the normalized root-mean-square error between the measured and estimated finger angular positions of the hand using different feature sets are shown. The x-axis letter labels represent the thumb, index, middle, ring, and little finger, while the numbers 1,2, and 3 are the CMC, MCP and PIP for the thumb and MCP, PIP and DIP for the rest of the fingers, respectively.

The results in Figure 5 show that using muscle activation input features not only parameterizes and considers EMD, but also gives better estimation result. The global estimation performance between three types of input: filtered EMG without EMD considerations, TD-based features and the proposed muscle activation inputs are shown. The figure shows the overall mean correlation coefficients and mean normalized root-mean-square error (NRMSE) of the actual and estimated joint kinematics of all the test data. In Figure 5(a), the proposed model using the muscle activation inputs, shown in red, performed better than other features shown in blue and green (averaging 7.38*%*±1.64*%* better than TD features and 13.13*%*±2.04*%* better than filtered EMG features). Significant differences were found when the correlation value using the muscle activation inputs was compared to the TD-based features (*P*<0.006) and to the filtered EMG inputs (*P*<0.001).

In Figure 5(b), the estimated finger kinematics using the muscle activation inputs across all DOFs had an average root-mean-square error of 11.53*%*±1.76*%*. Significant differences were also found when NRMSE using the muscle activation inputs was compared to the TD-based features (*P*(*R* 3,*L* 2,*L* 3)<0.05; *P*(*others*)<0.03) and to the filtered EMG inputs (*P*(*L* 3)<0.05; *P*(*others*)<0.01).

A three-way ANOVA testing the effects of different factors such as across different input features, across subjects and across finger DOFs showed significant differences in the correlations of the resulting estimation performance between the factor groups. Across the different input features used, the use of the proposed muscle activation features had significant differences, performing consistently better than other types of features used. Significant differences were also found between the different mean correlation coefficients across subjects and the finger DOF groups (*P*<0.001). Significant interactions were found for the Subject-Finger DOF and Subject-InputFeature pairs (*P*<0.001), while no significant interaction was found in the Finger DOF-Input Feature interaction (*P*=0.110). Tukey-Kramers’ comparison test found that the estimation performance among the three different input features used were different (correlation coefficient: muscle activation > TD-based > filtered EMG and NRMSE: muscle activation < TD-based < filtered EMG).

**Figure 6:**
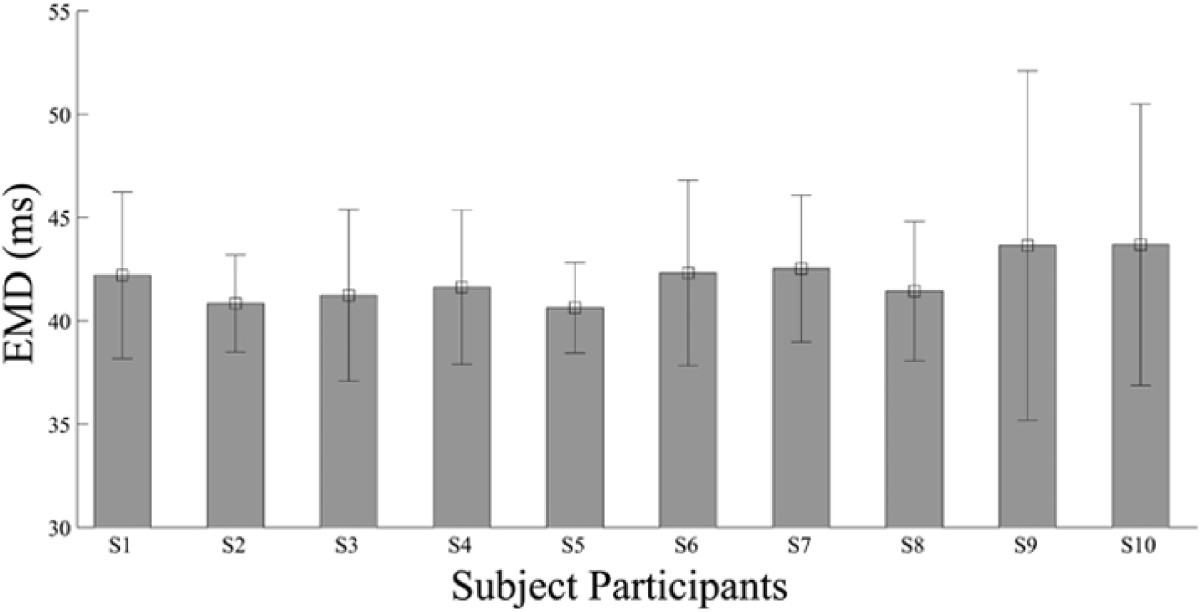
**The EMD parameters obtained across all the subject participants.** The electromechanical delay parameter obtained through optimization across different trials ranges from 39.6 ms to 75 ms, with a mean of 42 ms.

In Figure 6, the obtained EMD parameter across the 10 subjects in different experiment trials are plotted. The optimized EMD value ranged from 39.6 ms to 75 ms. No significant difference was found among the mean of the EMD values obtained across the 10 subjects (*P*=0.24). This supports our assumption that the obtained EMD across the subjects did not drastically change as the subjects tried to do the target tasks at constant velocity or at their normal and consistent pace across the trials. Obtaining an optimal value for the EMD using the optimization method described in the paper is important and can significantly improve the estimation performance compared to when no EMD is considered.

**Figure 7:**
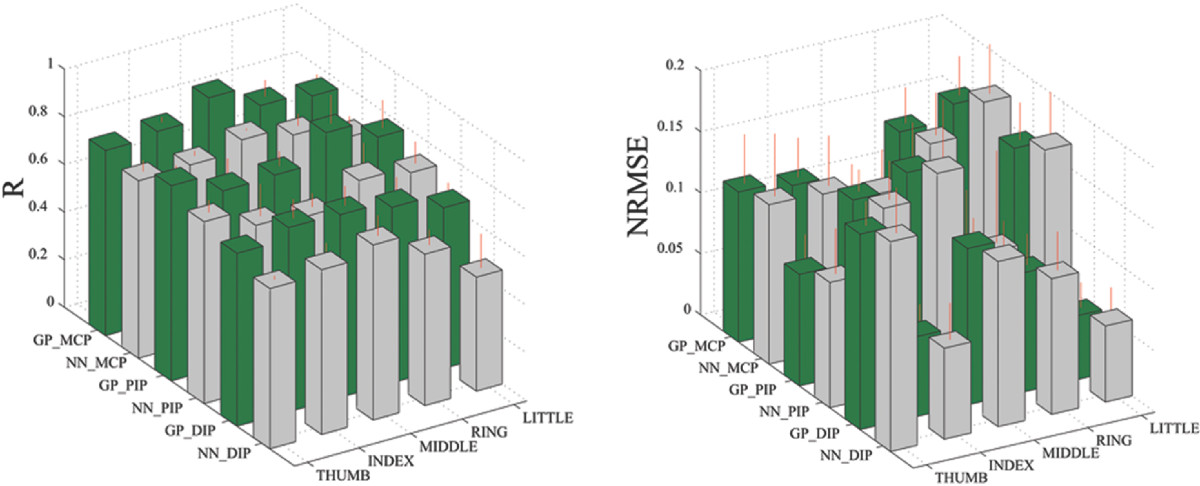
**Comparing between the use of NN and GP regressors.** The correlation coefficient (R) and the normalized root-mean-square error (NRMSE) of the measured and estimated finger DOFs are shown when the NN and GP regressors were used.

In Figure 7, the estimation performance is shown between the two regression methods used, namely using the NN and GP regressors. The estimation performance comparing the results in NN and GP regression averaged over 10 subjects are shown in this figure. For these results, the GP performed consistently better in all the subjects than the NN specially when training samples were sufficient. GP showed an average of 7.18*%* higher correlation performances than NN regression between the actual and estimated finger kinematics and when trained with 4800 samples. There was also significant differences in the obtained correlation coefficients between GP and NN regression (*P*<0.001). Overall, estimation of the MCP joint angles performed consistently better than the PIP and DIP estimation.

**Figure 8:**
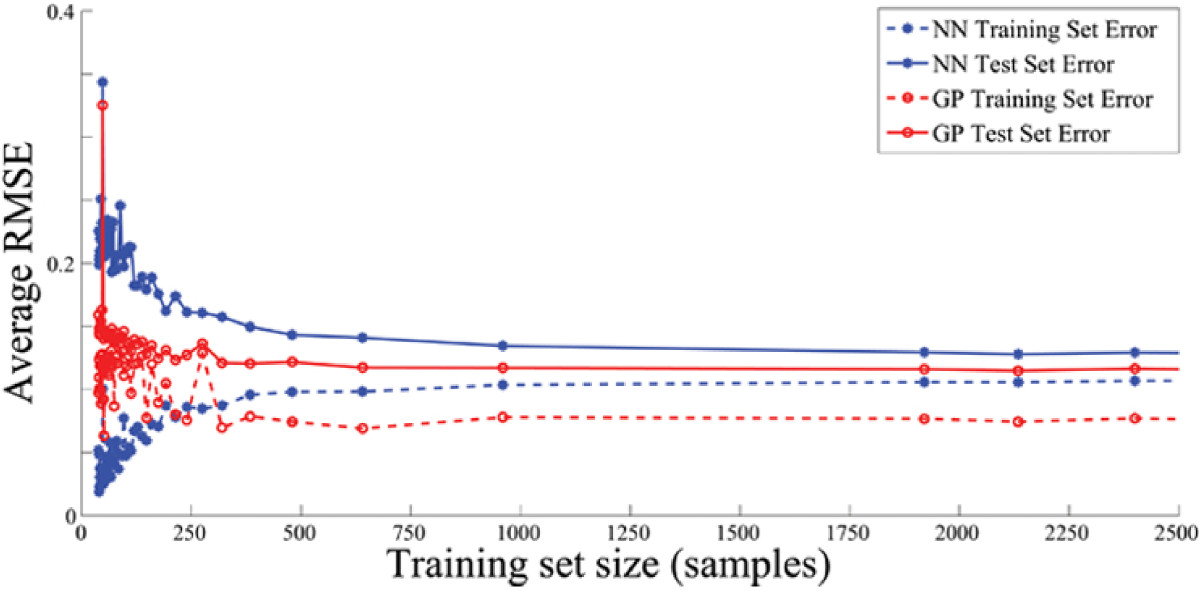
**Learning curves of the neural network and Gaussian Process Regressor.** The y-axis shows the average NRMSE while the number of training samples was varied. The number of test data samples remained fixed across all the subjects.

As the size of the training sample increases, NN performs better or much closer to GP with no significant increase in computation time, while GP computation suffers with the increase. Figure 8 shows the global performance of the estimators that we used, plotting the average RMSE of all the joint angles when the number of training samples was varied. As few as 250 samples for GP can give almost equal or even better performance as when more than 1800 samples are used to train a neural network. With more and more training samples available that captures more variability in the EMG and kinematics data, the neural network performs better reaching to the point where estimation performance is very close to GP as shown in Figure 4, where the estimation results showed the NN and GP performance over 4800 training samples.

## Discussion

This paper is the first to demonstrate the feasibility of estimating all finger joint kinematics using surface EMG that even considers electro-mechanical delay present in EMG-to-motion estimation applications and analysis. Compared to the use of pattern classification techniques used previously by many studies, we have presented results in doing simultaneous and proportional control of multiple finger DOFs comparing two different regression methods using EMG.

This paper also presented results taken from the smaller finger PIP and DIP joint angles, which have been rarely reported in any previous studies. Overall, our proposed method, which used the EMG-to-Muscle activation model, showed comparable, and in some instances, a more superior performance compared to that of the previous studies.

From the results of the ANOVA tests, we have shown that there are significant differences in mean estimation performance across difference factor groups such as across different types of input features used, across different subjects and across the finger DOFs. As mentioned previously, the use of the proposed muscle activation inputs gave a consistently better estimation performance compared to when other types of features were used. For the subject group used in this study, the 7 subjects’ estimation performances, both correlation coefficients and RMSE between the estimated and measured DOFs, were slightly, significantly better than the other 3 remaining subjects. It is not clear why some of the subjects performed poorly than the others, although the choice of random finger movements in the free movement task set across the subjects were different. Some of the subjects chose to do periodic flexion and extension movements in the free moving task while others chose to do more random, nonperiodic and more varying simultaneous and multiple finger movements. As for the differences across the finger DOF group, this can be attributed to the better performance achieved in estimating the MCP joint DOFs than in the PIP and DIP joint DOFs. The MCP DOFs have more independent movements than the PIP and DIP, which are more closely coupled and have dependent movements.

### On using the EMG-to-muscle activation model features

Processing the raw EMG signals into its muscle activation dynamics was straightforward. Training was fast and requires only a few parameters which is suitable for practical applications. For most trials, the EMD obtained ranged from 45 to 65 ms, suitably aligning the EMG onset to the motion data. We hypothesize that this model works very well for motion with constant velocities, as EMD has been known to change with the velocity and frequency of the task movement [18]. In the paper [27], the authors presented the time constants of the filters used in analyzing surface EMG, which ranged from 10 to 150 ms. It was also mentioned that the time constant should be changed adaptively to the data. In our method, the appropriate filter parameters, including the EMD in the muscle activation model were obtained through an iterative optimization procedure that minimized reconstruction error.

In our previous study, for periodic motion, the PIP and DIP angles followed movements similar to the MCP angles, but for random motions, it may totally differ. The input feature sets that we used do not give an explicit feature that relates the angles to one another [28]. However, compared to feature sets used by previous studies, the proposed set of muscle activation features performed better.

Furthermore, compared to reported EMD values in the other studies, the range obtained is comparatively smaller compared to those taken from the lower limb during cycling tasks [18] or from the upper limb during object-carrying tasks [3]. This can be attributed to observations such as the tasks involved in this experiment are faster, have smaller deviation in movement trajectories, and that the targeted muscles in the forearm are physically smaller. However, it is hypothesized that as the frequency or velocity of the finger movement tasks increases, then the EMD values may also significantly change.

### Neural network versus Gaussian process regression

Currently, there is no existing model that can best describe the relationship between EMG and finger joint kinematics. This is the main reason why we chose an artificial neural network and a Gaussian Process regressor, as these give a model-free approach in mapping the EMG signals to the corresponding finger kinematics.

Using artificial neural networks has been the primary choice in mapping the EMG to kinematics application, however, in this study we present the use of a nonparametric Bayesian approach through the use of a GP regressor. GP can give better estimation of the joint angles using fewer training samples as shown in Figures 7 and 8. This advantage is particularly important in not only reducing the amount of training time but in potentially reducing the amount of experiment protocol needed to capture large variations in the training data. In many myoelectric control strategies that are based on supervised learning, subjects have to retrain day after day as EMG signals are highly variable. With GP regressors, higher estimation accuracy compared to using neural networks can be achieved using fewer training data. Although not shown, using GP outperformed any neural network configuration, such as single output or multi-output network configurations [29], in the case of only few training samples available.

However we should point out that, though GP can handle missing data more readily than neural networks, the computation time becomes significantly higher in the former as the size of the training data increases. It took about 10 times longer to train the GP than the neural network. But with increasing computing capabilities of CPUs and computers, it will be but a matter of time before Bayesian regressors can be fully realized in practical applications. Also, in this study, the choice of covariance function was a standard Gaussian function. Other suitable choices for the covariance and mean functions may exist that can better improve the estimation performance, however, these have not been explored in this study. For this work, using GP regressors gave promising results in terms of getting better estimation using fewer training samples.

Also, in this study, we are estimating 15 finger joint kinematics simultaneously from eight muscle activation inputs. However, a dimensionality analysis on the hand kinematic data suggests that the effective dimension is less than the total DOFs available anatomically on the hand. By applying a Principal Component Analysis (PCA) on the finger kinematics data, the analysis showed that only the first 4 to 6 principal components explained the vast majority of the variance in hand posture. PCA was performed not only on the joint angular position data, but also on the joint angular velocities data because these are said to be more closely related to the motor command’s driving moment [30]. This is consistent with earlier studies, where it was shown that despite the hand having more than 20 DOFs, the effective dimensionality is much lower [31]. This can be attributed to factors such as mechanical constraints in the structure of the hand, high correlations of movements between joints and possibly the existence of synergies [31, 32]. However, the extent to which each of these DOFs is independently controlled during movement is still vague.

### Implementation and limitations

Most of the analysis has been done offline, however the proposed method is also suitable for real-time applications. In using the proposed muscle activation model, training and optimization is fast as there are only a few parameters needed in the transformation of the input features. Simultaneous and proportional estimation of all finger DOFs using the feedforward Neural Networks can be done real-time with delays of less than 100 ms. Though not reported in this study, a practical real-time application using the proposed method with the neural network in controlling a custom-built one-finger exoskeleton has been done in our previous study [33]. In that previous work, training was done using a mirror training scheme where the EMG data were obtained from a contra-lateral hand and were used to actuate the finger exoskeleton on the opposite hand.

The current subjects have been limited to healthy, able-bodied subjects to test the feasibility of our approach. This can be used as a benchmark for future implementation and validation for training amputees or subjects with hand impairments. The estimation of finger joint kinematics has also been confined to a static wrist and arm position. Changing the wrist’s position may influence finger joint estimation from EMG similar to those observed by Jiang et al. [34]. One possible solution is to increase the amount of training data by adding finger joint information at different positions of the arm and wrist. However, getting this amount of data may be impractical to apply in the real application setting. So there is a need to check if the GP can handle variations in the arm and wrist position. If dynamic arm and wrist position are to be considered, some form of hierarchical model may be considered. Currently, only the neural network has been fully tested on a real-time application. Other works are currently ongoing, which includes implementing other regression methods in real-time.

## Conclusion and future works

This work has presented an alternative and improved method in estimating simultaneous finger kinematics from EMG using a muscle activation model that parameterizes electromechanical delay (EMD), which has been observed by numerous investigators. Overall, our current method captures the general trend of finger movements and is able to estimate multiple finger DOFs with usable and reasonable accuracies. Due to the high variability in hand anatomy and internal control strategies, up to this date, no existing biomechanical model can capture the complex movement of the hand. Thus, using a model-free approach such as an artificial neural network or a nonparametric Gaussian Process is suitable in estimating finger kinematics from muscle activation inputs. Though neural networks are fast and perform robustly well when the training data is sufficient, using a Gaussian Process regressor gives better performance when the training samples are small. This shows much promise in being able to reduce the amount of experiment training protocols substantially and can work better than using neural networks.

The approach proposed in this paper presents a practical solution for a myoelectric control strategy for proportional and simultaneous control of multiple DOFs in robotic hand prostheses and finger exoskeletons. Though our method validates the feasibility of position-based control from surface EMG, manipulating small objects or doing skillful tasks are much more complex. We need to investigate further how we can deal with overall variations in hand movements such as dealing with the effects of different positions of the arm or wrists. The future work of our study involves investigating muscle activation patterns for object manipulation and integrating a torque-based control strategy that can further improve current state-of-the-art myoelectric control strategies.

## Authors’ original submitted files for images

Below are the links to the authors’ original submitted files for images. Authors’ original file for figure 1 Authors’ original file for figure 2 Authors’ original file for figure 3 Authors’ original file for figure 4 Authors’ original file for figure 5 Authors’ original file for figure 6 Authors’ original file for figure 7 Authors’ original file for figure 8

## Abbreviations

EMG: Electromyography
EMD: Electro-mechanical delays
ANN: Artificial neural network
GP: Gaussian process
MCP: metacarpophalangeal
PIP: Proximal interphalangeal
DIP: Distal interphalangeal
DOF(s): Degree of freedom(s)
MVC: Maximum voluntary contraction
TD: Time domain
MAV: Mean of the Absolute value
WL: Waveform length
WL: Willison amplitude
VAR: Variance
NN: Neural network
NRMSE: Normalized root-mean-square error
ANOVA: Analysis of variance
IP: Interphalangeal
CPU: Central processing unit
PCA: Principal component analysis.
