# Supplementary figures and images for: Continuous and simultaneous estimation of finger kinematics using inputs from an EMG-to-muscle activation model
Source: J Neuroeng Rehabil. 2014 Aug 14;11:122. doi: 10.1186/1743-0003-11-122 (PMC4148535; doi:10.1186/1743-0003-11-122)

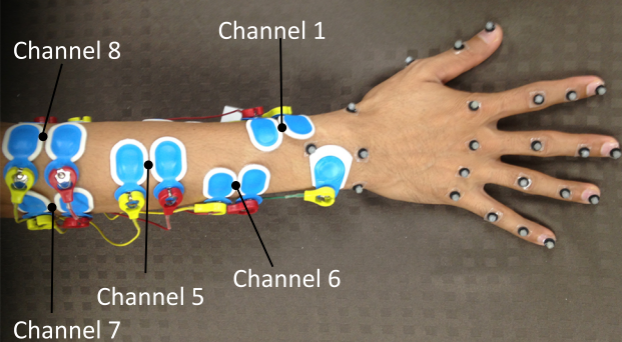

Channel 1

Channel 8

Channel 6

Channel 5

Channel 7

Supplement: Supplementary file 1 — Authors’ original file for figure 1 [file 12984_2013_645_MOESM1_ESM.pdf]

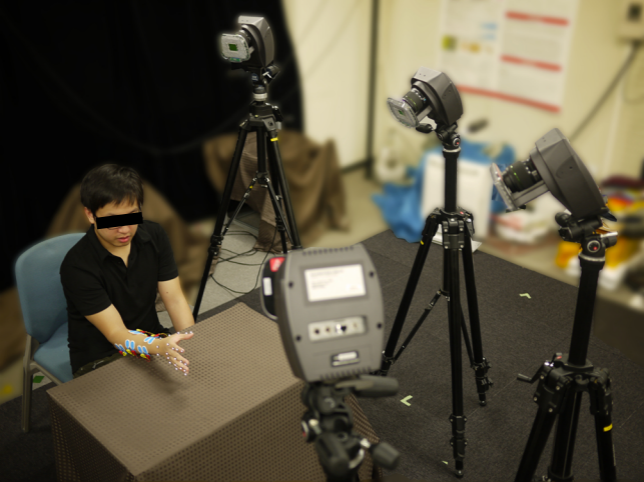

Supplement: Supplementary file 2 — Authors’ original file for figure 2 [file 12984_2013_645_MOESM2_ESM.pdf]

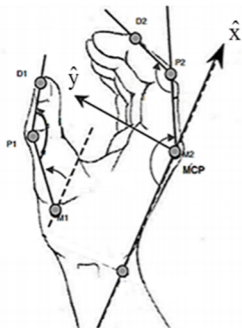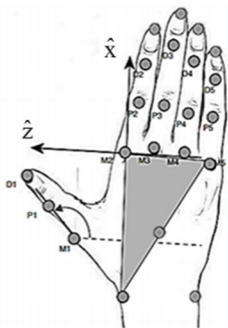

(a)

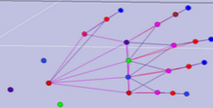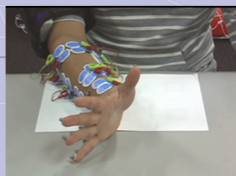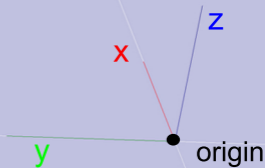

(b)

Supplement: Supplementary file 3 — Authors’ original file for figure 3 [file 12984_2013_645_MOESM3_ESM.pdf]

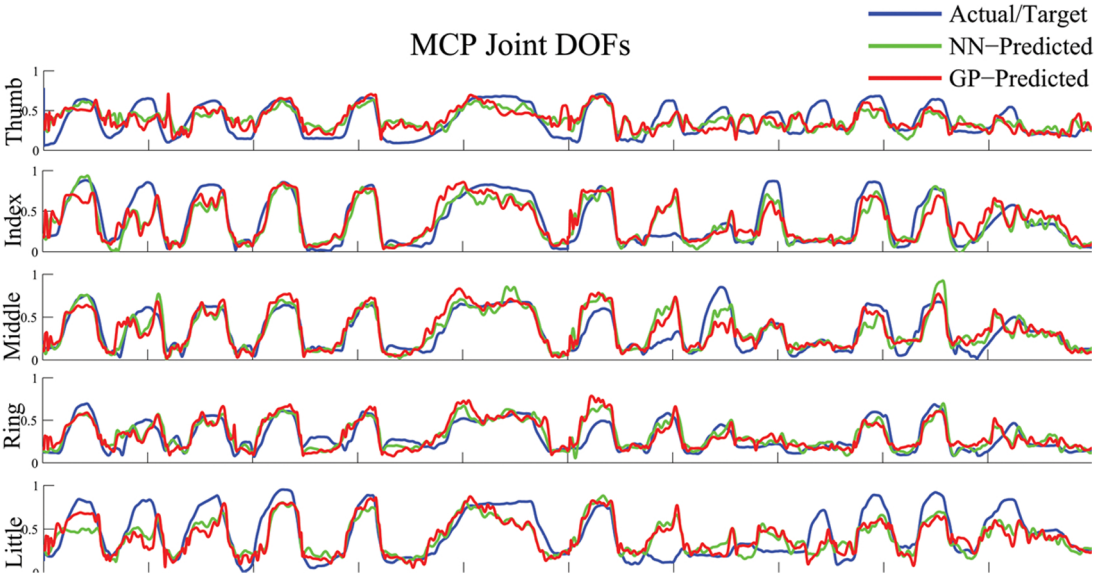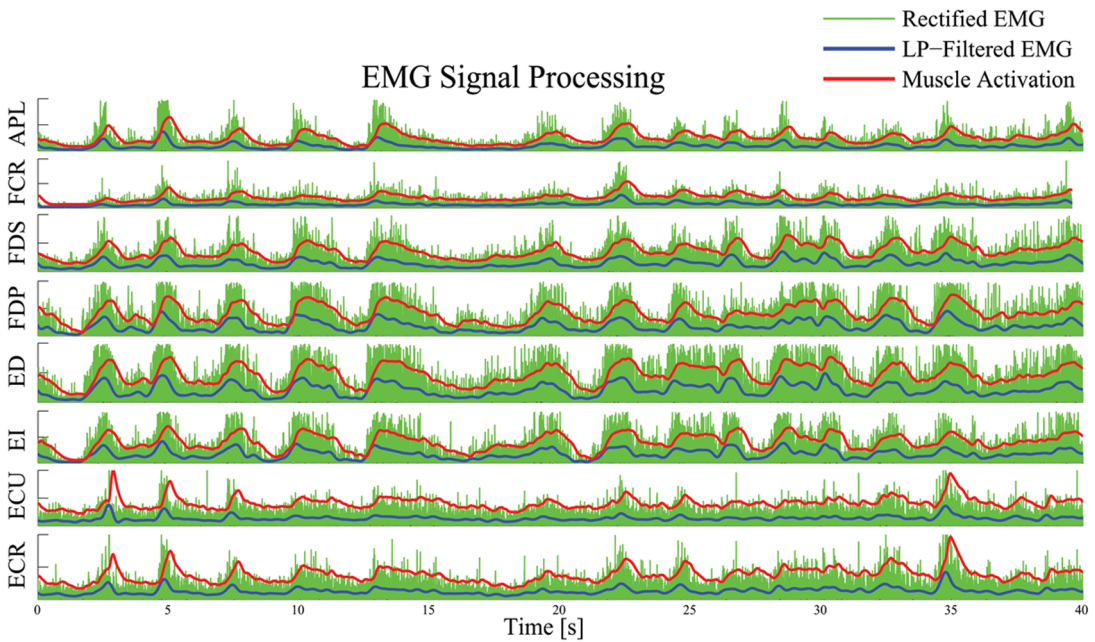

Supplement: Supplementary file 4 — Authors’ original file for figure 4 [file 12984_2013_645_MOESM4_ESM.pdf]

# Mean Correlation and NRMSE of the Estimated Finger DOF

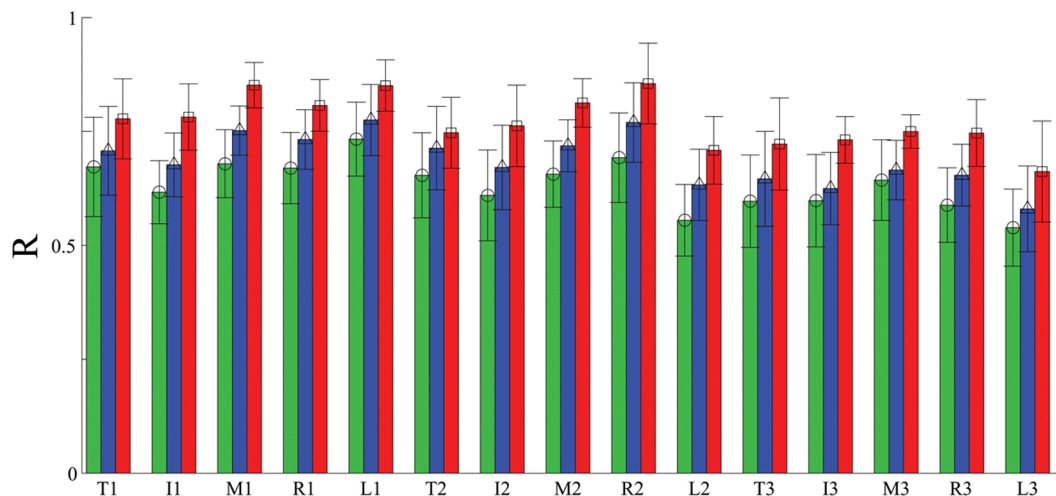

(a)

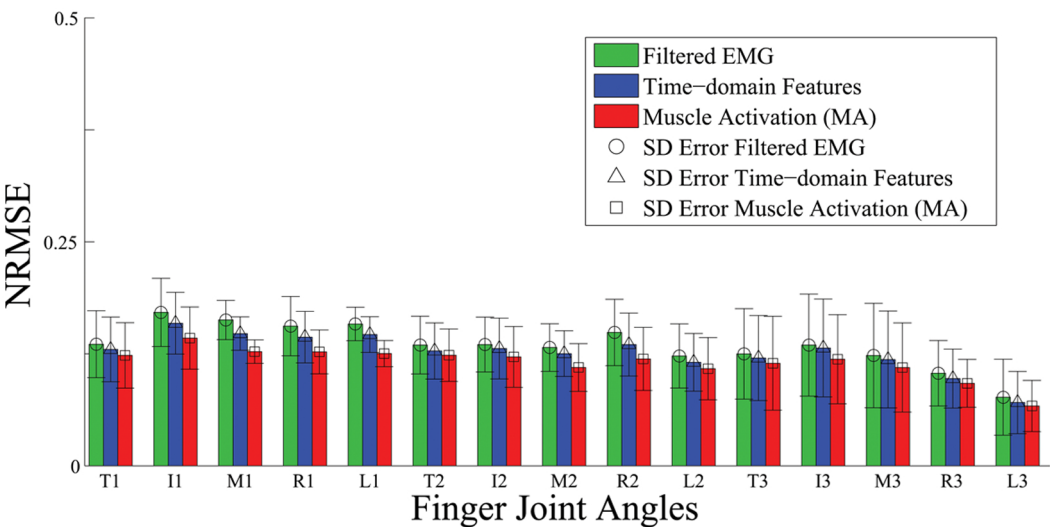

(b)

Supplement: Supplementary file 5 — Authors’ original file for figure 5 [file 12984_2013_645_MOESM5_ESM.pdf]

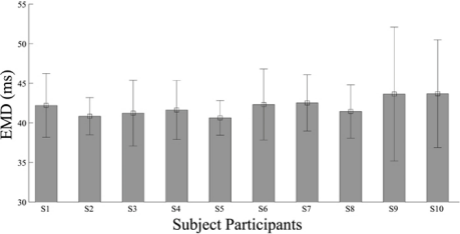

Supplement: Supplementary file 6 — Authors’ original file for figure 6 [file 12984_2013_645_MOESM6_ESM.pdf]

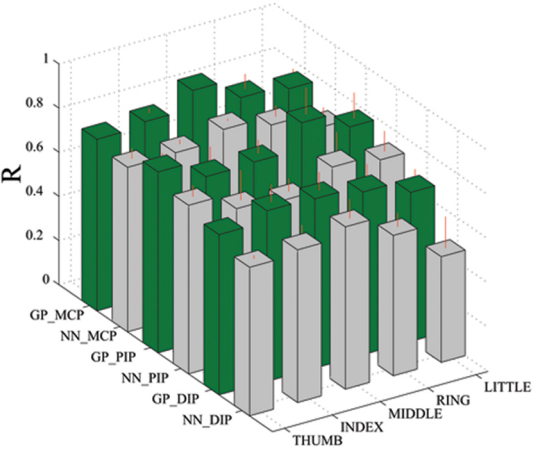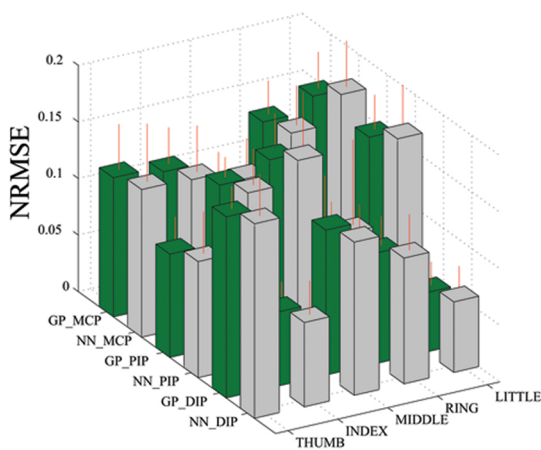

Supplement: Supplementary file 7 — Authors’ original file for figure 7 [file 12984_2013_645_MOESM7_ESM.pdf]

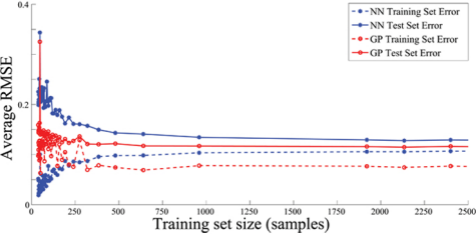

Supplement: Supplementary file 8 — Authors’ original file for figure 8 [file 12984_2013_645_MOESM8_ESM.pdf]
